# Capacitive Sensing for Non-Invasive Breathing and Heart Monitoring in Non-Restrained, Non-Sedated Laboratory Mice

**DOI:** 10.3390/s16071052

**Published:** 2016-07-07

**Authors:** Carlos González-Sánchez, Juan-Carlos Fraile, Javier Pérez-Turiel, Ellen Damm, Jochen G. Schneider, Heiko Zimmermann, Daniel Schmitt, Frank R. Ihmig

**Affiliations:** 1 Fraunhofer-Institut fuer BiomedizinischeTechnik (IBMT), Sulzbach/Saar 66280, Germany; cgonzalezs90@gmail.com (C.G.-S.); heiko.zimmermann@ibmt.fraunhofer.de (H.Z.); daniel.schmitt@ibmt.fraunhofer.de (D.S.); frank.ihmig@ibmt.fraunhofer.de (F.R.I.); 2 ITAP—Universidad de Valladolid, Paseo del Cauce 59, Valladolid 47011, Spain; turiel@eii.uva.es; 3 Luxembourg Centre for Systems Biomedicine, University of Luxembourg Esch-sur-Alzette L-4362, Luxembourg and Internal Medicine II, Saarland University Medical Center, Homburg 66421, Germany; ellen.damm@uni.lu (E.D.); jochen.schneider@uni.lu (J.G.S.); 4 Molecular and Cellular Biotechnology/Nanotechnology, Saarland University, Saarbruecken 66123, Germany

**Keywords:** non-invasive sensor, capacitive sensors, physiological signals in mice, stress in mice

## Abstract

Animal testing plays a vital role in biomedical research. Stress reduction is important for improving research results and increasing the welfare and the quality of life of laboratory animals. To estimate stress we believe it is of great importance to develop non-invasive techniques for monitoring physiological signals during the transport of laboratory animals, thereby allowing the gathering of information on the transport conditions, and, eventually, the improvement of these conditions. Here, we study the suitability of commercially available electric potential integrated circuit (EPIC) sensors, using both contact and contactless techniques, for monitoring the heart rate and breathing rate of non-restrained, non-sedated laboratory mice. The design has been tested under different scenarios with the aim of checking the plausibility of performing contactless capture of mouse heart activity (ideally with an electrocardiogram). First experimental results are shown.

## 1. Introduction

Animal testing has played and plays a vital role in biomedical research. Its use has revealed the therapeutic effectiveness and efficiency of new techniques and substances, chemicals and medicines, as well as their toxicity or safety, without putting at risk the lives and safety of humans.

Despite the undeniable benefits that these techniques provide to mankind, we need a moral code that minimizes the impact of research on animals. This need has been defended by sectors both within and outside the scientific community since the 19th century, and has progressed to current practice, summarized in the “proposal of the three Rs”: replacement, reduction, and refinement. This proposal was published by Russell and Burch in 1959 [1], and is based on three pillars that scientific experimentation must comply with whenever possible:

- Replacement: refers to methods which avoid or replace the use of animals in an area where animals would otherwise have been used. This includes both absolute replacements (i.e., replacing animals with inanimate systems, such as computer programs) and relative replacements (i.e., replacing more sentient animals, such as vertebrates, with animals that current scientific evidence indicates have a significantly lower potential for pain perception, such as some invertebrates).
- Reduction: refers to any strategy that will result in fewer animals being used to obtain sufficient data to answer the research question, or in maximizing the information obtained per animal.
- Refinement: refers to the modification of husbandry or experimental procedures to minimize pain and distress, and to enhance the welfare of an animal used in science from the time it is born until its death.

Despite these efforts, millions of animals are needed each year in laboratories around the world. In the European Union, nearly 11.5 million laboratory animals were used in 2011, about 75% of which were rodents [2].

In research, it is important to minimize all external influences that could undesirably modify the response of laboratory animals, bringing the results closer to the reality of the problem. By having better models we can reduce the number of subjects used during tests.

One parameter that may introduce discrepancies is stress [3]. Reducing stress is important for improving the results of research, as well as for increasing the welfare and quality of life of laboratory animals. Any change in the environmental conditions of an animal can induce stress, transport being of particular significance, especially for laboratory animals. In the case of genetically modified animals, transport stress is of even greater importance, as specialized companies make shipments of their specimens on request to laboratories around the world, with a consequent increase in transport time that exacerbates the possible symptoms.

There is evidence that transport produces high stress levels. An assessment of stress during handling and transport of animals is presented in [4]. This stress is not confined to the transported animal, but it can also affect their offspring [5,6]. Different parameters have been used to assess stress in animals, such as: cortisol, corticosterone and flucose, food and water consumption and weight loss [7]. After high stress levels, the mice require much time for the heart rate (HR) and breathing rate (BR) physiological signals to return to values within the normal levels.

For example, the need for 24 to 48 h of rest for the immune system and corticosterone levels to stabilize after transport has been described [8,9]. Another study that monitored stress indicators based on animal behavior and the corticosterone level after transportation found that mice were not completely acclimated after three to four days [3,10].

Aiming to minimize stress, some factors must be taken into account:

- Animal health (especially their ability to travel).
- Design and materials of containers, including loading and unloading as well as measures to inspect the condition of the animal during transport.
- Number of animals in each container and the allocated space for each animal.
- Environmental conditions inside the container.
- Quality and quantity of substrate or material covering the bottom as well as food and water (or additional liquid source).
- Travel time.
- Number of trips or changes between vehicles.
- Recovery of the animal after the trip.

We believe it is of great importance to develop non-invasive techniques for monitoring physiological signals during the transport of laboratory animals. This allows the gathering of information about the conditions of transport with an aim to improve these conditions according to the principle of the three Rs. This will improve the quality of life of the animal, and will also improve the performance that this animal provides for science, further decreasing the number of animals required for research.

This paper presents the developed approach based on EPIC (electric potential integrated circuit) sensors to measure electric potential for capturing physiological signals in mice, with emphasis on non-invasive and contactless techniques.

## 2. Experimental Section

### 2.1. StressMeasurement in LaboratoryAnimals

One commonly used method for monitoring the level of stress in laboratory animals during transport is based on heart rate variability (HRV). This term is defined as beat-to-beat changes in heart rate or variations of the RR intervals (time between two R waves in an electrocardiogram) in consecutive cardiac cycles. HRV provides a more accurate measurement of stress than those obtained simply by observing cardiac rhythm, because the cardiac rhythm can vary greatly from one animal to another, or depend on physical activity. Measuring inter-beats intervals (IBI) and HRV are promising approaches for evaluating stress in animals [11]. The importance of HRV as a potential marker of stress and health is shown in [12].

However, acquisition of the heart rate is not a trivial task in small laboratory animals. Invasive techniques are often used, based on implantable electrodes placed inside the animal that might transmit information through a radio link [13,14]. These systems provide a complete and detailed electrocardiogram (ECG) signal, but require hard work and dedication by the researcher and problems may arise necessitating post stress rest or increasing the risk of death of the animal.

While there are non-invasive ECG acquisition techniques for rodents, these often require training of the animal and placing it in a certain position or device. Capture of ECG signals with wet electrodes is often associated with discomfort because the electrodes require skin preparation and the use of gels. These gels tend to lose moisture over time with the consequent loss of ECG signal. Thus, these techniques are not feasible for long-term continuous monitoring.

The development of an ECG monitoring system using EPIC sensors that do not require the preparation of the skin or direct contact with the subject would be of great help in improving the usability of these systems.

### 2.2. Capacitive Sensors

A particular type of dry electrode, first developed by Lopez and Richardson [15], is known as a capacitive or insulated electrode. These electrodes require no ohmic contact with the body since it acts as a simple capacitor placed in series with the skin, so that the signal is capacitively coupled. The received signal can be connected to an operational amplifier and then to standard instrumentation.

The use of a dielectric material in good contact to the skin results in a fairly large coupling capacitance, ranging from 300 pF to several nano-farads. As a result, a system with reduced noise and appropriate frequency response is readily achievable using standard high-impedance FET (field-effect transistor) amplifiers. 

While wet and dry electrodes require physical contact with the skin to function, capacitive electrodes can be used without contact, through an insulating layer such as hair, clothing or air. These contactless electrodes have been described generally as simple capacitive electrodes, but in reality there is also a small resistive element, since the insulation also has a non-negligible resistance. Signals coupled via contactless electrodes can contain unacceptable levels of noise, requiring a suitable design of other elements to increase the input impedance and neutralize the parasitic input capacitance. One of the major drawbacks of these electrodes is their sensitivity to movements, which along with the high settling time can render these systems inadequate in certain scenarios.

Capacitive sensors have been used to measure heart rate in humans via either direct skin contact or through one and two layers of clothing with no dielectric gel and no grounding electrode [16,17,18]. These sensors have also been used to monitor human respiratory rate using a conductive textile-based wearable sensor [19,20]. High impedance electric potential sensors have been used in humans to measure breathing and heart signals [21,22,23]. However, we have found no report of the use of capacitive sensors for monitoring heart rate in mice during transport.

**Figure 1:**
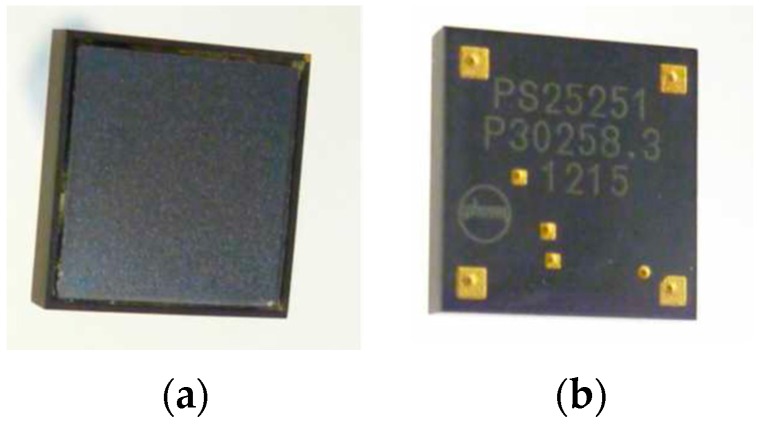
PS25251 EPIC sensor, front (**a**) and rear (**b**) views.

In our experiments with mice, we have used the PS25251 EPIC capacitive sensor (see Figure 1) that incorporates a number of tools to enhance their characteristics, allowing measurement of the electric potential. Its main features are: ultra-high input resistance, typically 20 GΩ, dry-contact capacitive coupling, input capacitance as low as 15 pF, lower −3 dB point typically 200 mHz and upper −3 dB point typically 10 kHz. It operates with bipolar power supply from ±2.4 V to ±5.5 V.

### 2.3. Capture of Physiological Signals in Rodents

A system for simultaneously monitoring the heart rate and breathing rate of anesthetized mice using a piezoelectric transducer is proposed in [24]. Noncontact monitoring of cardiorespiratory activity by electromagnetic coupling has been investigated in humans, by using two measurement modalities: the capacitive coupling path and the inductive coupling path (magnetic induction monitoring) [25,26]. In this paper, we propose to use capacitive sensing for non-invasive breathing and heart monitoring in non-restrained, non-sedated laboratory mice.

This research focuses on the capture of physiological signals in laboratory animals, particularly C57BL6/J/LDLR^−/−^ strain mice. Animal experiments were approved by the Saarland University Medical Center animal experimentation office (animal protocol 30/2012).

The aim of the proposed experiments was the contactless capture of mouse heart activity (ideally ECG), using the EPIC sensors, and taking into account the following technical difficulties:

- Small amplitude of signal due to the small size of the mouse heart.
- The impossibility of using direct methods of noise reduction, such as the grounding of the mouse or its connection to the DRL (driven right leg) signal.
- Low capacitive coupling with the sensor when using contactless techniques.

Because of these limitations, we have conducted experiments to determine the feasibility of this technique, and also to evaluate other options, such as the use of techniques with contact between the animal and sensors. Different configurations have been used depending on the type of experiment, but the hardware and software was always similar.

#### 2.3.1. Sensor Instrumentation Circuit

Usually, the weak cardiac signal appears as the differential signal between two electrodes. To remove common mode noise we used an instrumentation amplifier, INA128 (Texas Instruments, Dallas, TX, USA), which has high common mode rejection, low power consumption and low price.

The sensors require a power supply voltage of ±5.5 V maximum, which is also suitable for the instrumentation amplifier. Batteries were used as power supply in order to reduce the environmental electrical noise.

**Figure 2:**
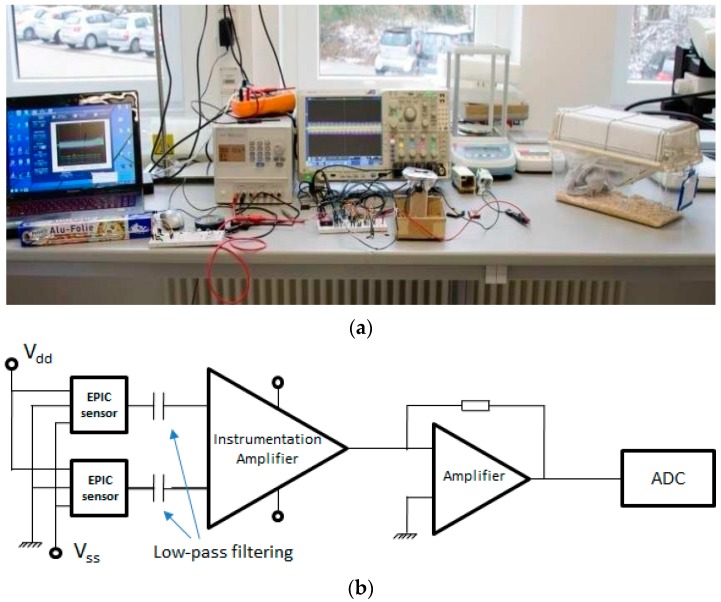
Experimental setup used to capture physiological signals in rodents (**a**).From left to right: Laptop for signal storage and processing, power supply, oscilloscope to capture signals, the instrumentation circuit, and a cage. Instrumentation circuit diagram implemented for this experimental setup (**b**).

From the pre-amp, the signal was fed to a high-pass first-order filter and amplified by one section of an OPA2131 operational amplifier (Texas Instruments), captured by a digital oscilloscope (MDO4104B-6, Tektronix, Beaverton, OR, USA) and stored on a PC. Figure 2a shows the experimental setup used for the acquisition of physiological signals in rodents. Figure 2b shows the instrumentation circuit diagram implemented for this experimental setup. 

Figure 2b shows the first section (on the left) with EPIC sensors and powering (Vdd = 5 V and Vss = −5 V). The second section (in the middle) includes the instrumentation amplifier, common-mode noise rejection and first amplification. The final section includes a second amplification and analog/digital converter (ADC).

#### 2.3.2. Signal Processing

We used MATLAB software (version R2014b) to implement a series of algorithms to improve the quality of the stored signal. We routinely used a notch filter to remove 50 Hz noise (and its multiples) and a low-pass filter. This was followed by a polynomial spline fit and subtraction from the original signal. If the signal was still noisy, we used a 5th order polynomial Savitzky-Golay filter, or generalized moving average to smooth it. Time and frequency domain plots were made after each one of these filters.

### 2.4. Platforms Developed for Capturing Physiological Signals in Rodents

Initial experiments required minimizing the movement of the mouse during the capture of physiological signals. Thus, we developed three platform systems to carry out tests. In Table 1, we summarize key aspects of the three systems.

We initially tested plastic tubes (system A) in which holes were made to ensure adequate airflow to allow for normal breathing. Two sensors, one on each side, were placed on the outside, with the animal in between. Finally, the plastic tube was surrounded with grounded aluminum foil in an attempt to reduce electrical noise picked up by the sensors.

**Figure 3:**
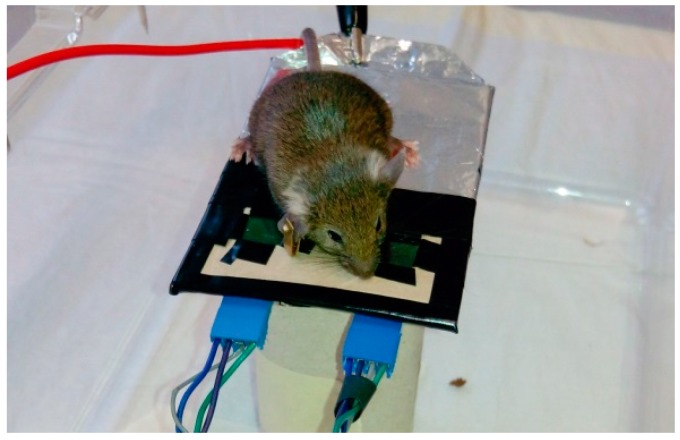
Platform (system B) on which the mouse is placed. Two EPIC sensors and the grounding can be distinguished.

Several experiments were performed, with a different approach, using the small platform (system B) shown in Figure 3.There are two EPIC sensors on the platform and the mouse is placed over it. It did not move from the area where the sensors are positioned.

**Figure 4:**
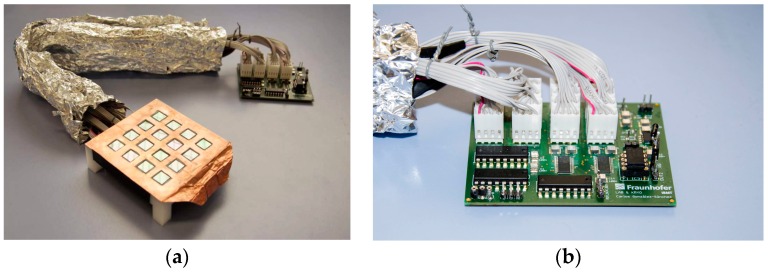
(**a**) Matrix of EPIC sensors (system C) used for physiological signals capturing in mice; (**b**) Self-designed PCB used to control the matrix of EPIC sensors.

Due to the difficulty of getting two of the mouse legs placed exactly over the two sensors, we proceeded to design the matrix of sensors (system C) shown in Figure 4a, that can accommodate 16 EPIC sensors arranged in a 4-by-4 matrix. After placing the sensors and making the necessary connections, the top was covered with an adhesive copper tape except, obviously, the areas where the sensors are located.

With the aim of improving the connection and control of this matrix of sensors, a printed circuit board (PCB) was designed and built (see Figure 4b). It contains an interface for connection and power management for up to 16 EPIC sensors, capable of reducing power consumption by modifying the number of sensors powered at a time, also ensuring adequate flexibility for future developments. This board also allows the user to select the pair of sensors to be used for the output signal and to calculate the DRL signal.

## 3. Results and Discussion

### 3.1. Test Trial 1: Plastic Tube Platform (System A)

**Figure 5:**
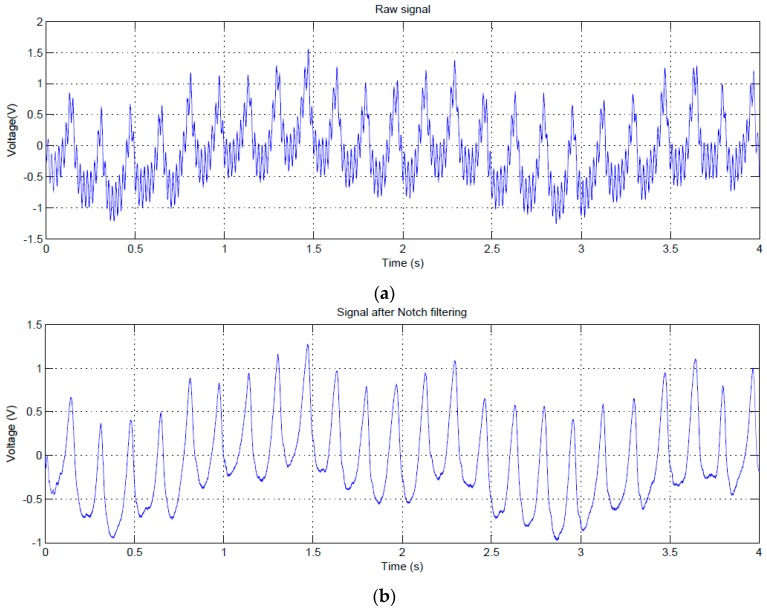
Test trial 1: signal captured with mouse placed inside a plastic tube (**a**); Signal after notch filtering at 50 Hz (**b**).

The mouse was placed inside a plastic tube with holes to ensure adequate airflow for normal breathing. EPIC sensors were placed on the sides of the animal and the tube was surrounded with aluminum foil connected to a ground (GND). The signal captured using this system is shown in Figure 5a. After applying the notch filter to the original signal, the signal shown in Figure 5b is obtained.

**Figure 6:**
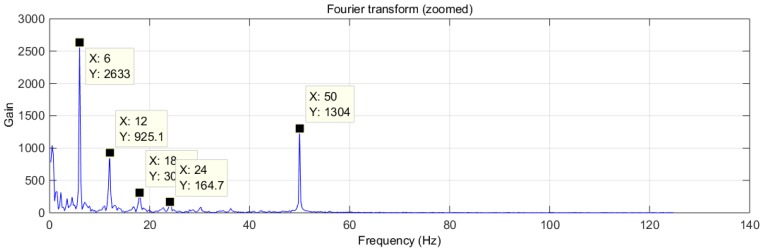
Test trial 1: transformed signal in the frequency domain.

The signals of Figure 5 show a clear periodic pattern. To study its characteristics we analyzed this signal in the frequency domain (Figure 6).

Figure 6 shows a significant component at 50 Hz, corresponding to electrical noise. However, there is an interesting regular arrangement at 6, 12, 18, 24, 30 Hz and so on (6 Hz multiples) of decreasing power. This was initially assumed to be electrical noise of unknown origin and its harmonics that the captured signal shows, but further tests discarded this possibility, since this signal was captured only when the mouse was present. 

An algorithm was used to locate the peaks (in Figure 5) and calculate the average rate, obtaining a value of 364 peaks per minute. This value is too low to correspond to a heartbeat, because in mice heartbeat ranges from 310 to 840 beats per minute (bpm). Moreover, under stress conditions (mouse inside a plastic tube in a limited space and recently handled by a human), it is expected that these values would be even higher.

The normal breathing rate in relaxed mice, however, usually ranges from 80 to 230 breaths per minute. Therefore, our first hypothesis was to associate the signal of Figure 5 (bottom) to a mouse breathing signal, probably produced by the distance variation between the sensor and the mouse’s body due to its chest movements during inspiration and expiration. Accordingly, we performed more tests using contact techniques to see if, in this way, it was possible to capture any kind of cardiac signal, using GND and DRL grounding techniques.

### 3.2. Test Trial 2: Platform (System B) with Sensors and GND Grounding

**Figure 7:**
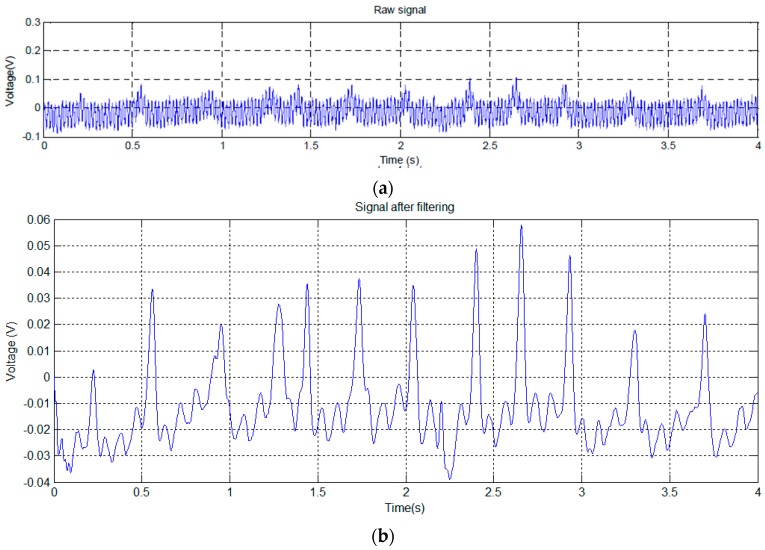
Test trial 2: time signal without any filtering (**a**); Signal after notch filtering at 50 Hz (**b**).

In this test, the mouse was placed on a small raised platform, which carried two EPIC sensors. The signal shown in Figure 7a has been obtained from the mouse with one paw in contact with one of the EPIC sensors, and the other in contact with the grounding (GND).

Since the (mainly 50 Hz) noise in Figure 7a masks the detailed signal characteristics, we used a notch filter to obtain the signal shown in Figure 7b.

**Figure 8:**
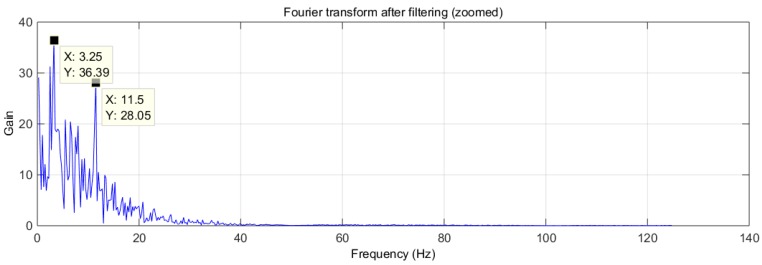
Test trial 2: transformed signal in the frequency domain.

The signal of Figure 7b shows clear periodic variations. Figure 8 shows the transformation to the frequency domain.

The signal shown in Figure 7b clearly shows several large peaks, similar to those shown in Figure 5b, produced by the breathing of the mouse. Its rhythm has been calculated using peak detection in the signal, resulting in 196 breaths per minute, which fits perfectly with one of the dominant frequencies of the spectrum (see Figure 8), located at 3.25 Hz (195 min^−1^). There is a second peak in Figure 8 at 11.5 Hz (690 bpm). Although high, this is within the normal heart rate range for mice.

**Figure 9:**
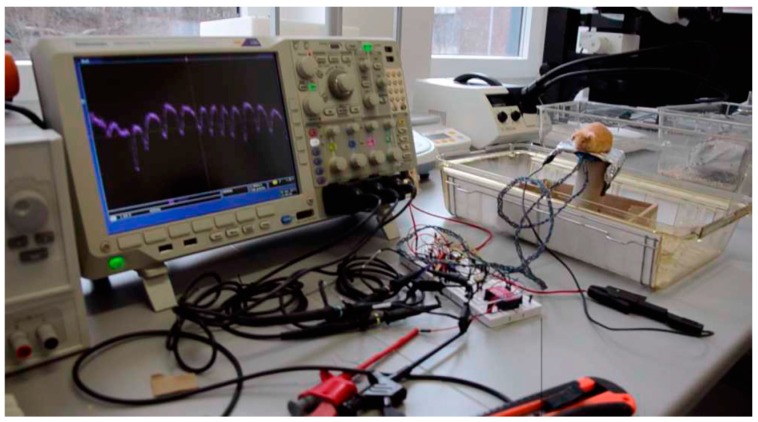
Still frame from one of the captured videos to verify the correlation between the captured signal and the breathing rate.

To check the correspondence of the strongest signal (lower frequency) with the respiratory rhythm of the mouse, a video camera was used to record both the movement of the mouse and the captured signal (Figure 9). 

**Figure 10:**
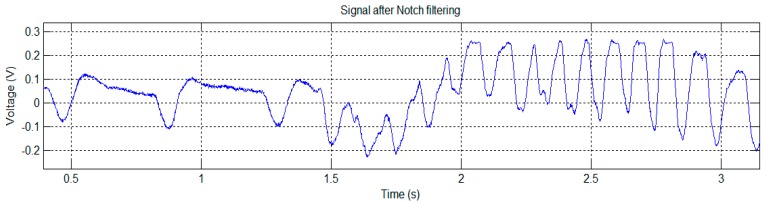
Mouse respiratory signal showing a sharp rate change due to sniffing.

Figure 10 shows the signal captured. This signal is related to the respiration of mouse, since it reflects the movements of the mouse’s chest. It can be seen that the frequency of this signal increases for a short time when the mouse starts sniffing. This signal shows the sudden change in the breathing pattern, just as the sniffing occurs. As shown in Figure 10, the mouse’s respiratory rate changes very abruptly, from 143 to about 465 breaths per minute. The signal becomes saturated briefly, but the frequency is still recognizable.

### 3.3. Test Trial 3: Platform (System B) with Sensors and DRL Grounding

**Figure 11:**
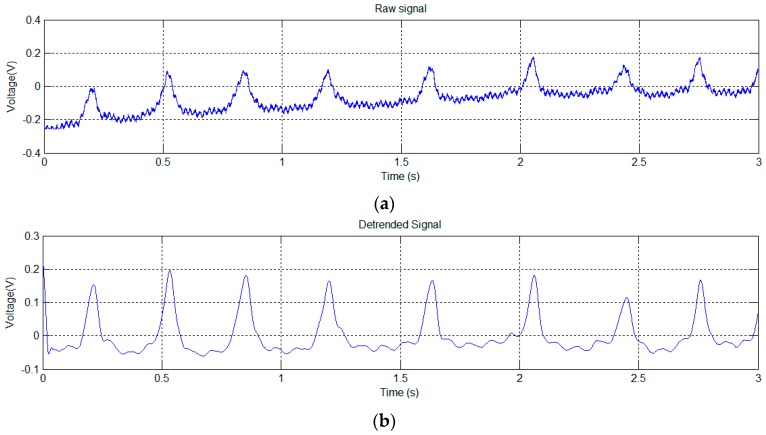
Test trial 3: signal captured using an elevated platform without processing (**a**) and after filtering out the average trend (**b**).

In this trial, we use the DRL signal, instead of the GND, as a grounding method. In the tests, one paw of the mouse was in contact with one of the EPIC sensors. The mouse’s captured signal is shown in Figure 11a. This signal is similar to the one shown in the previous sections, although showing a much lower level of electrical noise at 50 Hz than in the previous case, probably due to the greater effectiveness of the DRL signal. After filtering and processing we got the signal shown in Figure 11b.

In Figure 11 we can see again two main components in the signal, just as in the previous case. The smaller-magnitude signal can be distinguished better in the valleys of the greater-magnitude component. Their frequencies have been calculated as 165 min^−1^ for the largest component, and 687 min^−1^ for the smaller, similar to those shown in test trial 2, and again attributed to respiratory and heartbeat processes, respectively.

**Figure 12:**
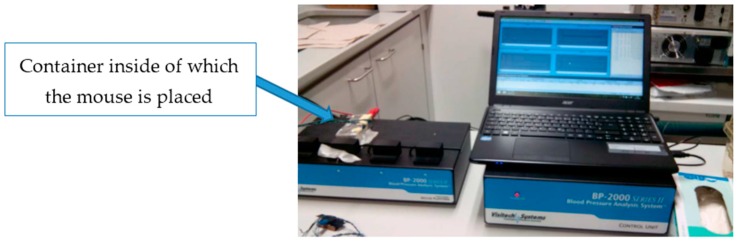
The BP-2000 blood pressure acquisition system in mice.

Making use of video recordings, we proved our hypothesis that the captured signal of greater magnitude corresponded to the respiratory rhythm. In order to get a reference value for the pulse in mice and compare it to the value obtained using EPIC sensors, we used a Visitech Systems BP-2000 commercial system (Figure 12). This device performs an analysis of the blood pressure in rodents using non-invasive techniques. Specifically, it uses small pliers that are placed on the animal’s tail to measure the amount of light absorbed, while keeping the animal inside small opaque containers that restrain its movement and keep the animal from escaping. Variations in the signal correspond to variations in size of the blood vessels due to heart movement (known as transmission photo-plethysmography). Variations in blood pressure (systole-diastole) can be inferred from these data and, hence, heart rate. The purpose of these measurements was the acquisition of a baseline heart rate signal, obtained under similar conditions to the ones used in our experiments, which we used to compare with our own signal.

**Figure 13:**
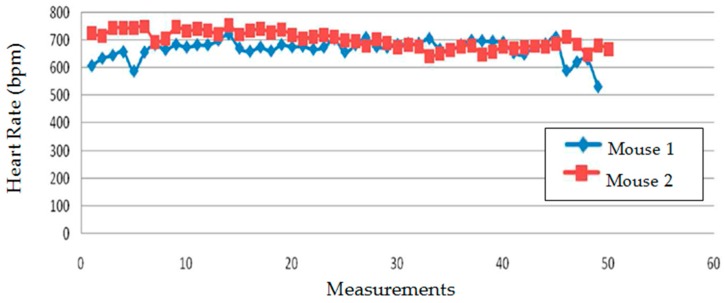
Heart rate values obtained from photo-plethysmography measurements in two mice.

Using this commercial system, 50 heart rate samples were captured in two mice, being the average values 668 and 701 bpm, respectively, as is shown in Figure 13. The value of 687 bpm which we got from the analysis of Figure 11 is therefore within the range obtained with the BP-2000 system.

### 3.4. Test Trial 4: Matrix of EPIC Sensors (System C) with Connection to GND, Two Pawsin Contact with the Sensors

**Figure 14:**
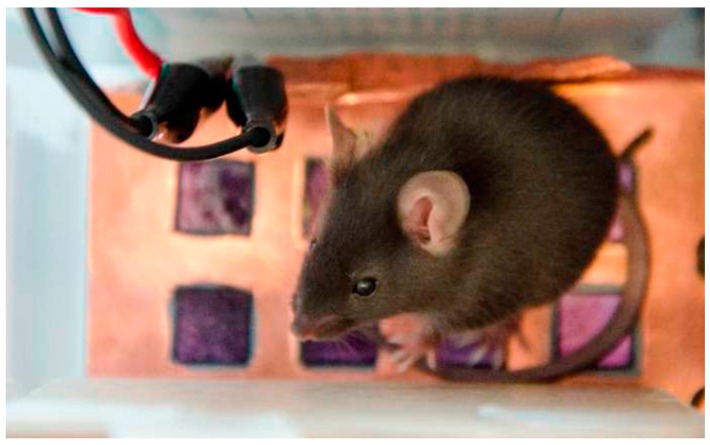
Test trial 4: mouse placed over the 4-by- 4 EPIC sensors matrix.

Because of the interesting results obtained when the animal had a leg in contact with the sensor, we used the 4-by-4 matrix of EPIC sensors (system C in Table 1), in which the distance between sensors had been kept as small as possible (16.8 mm pitch). The matrix is shown in Figure 14, where the grounding and the sensors are clearly visible.

**Figure 15:**
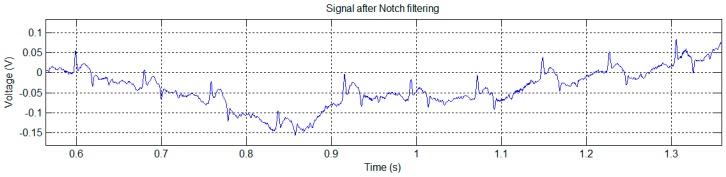
Test trial 4: signal obtained after notch filtering the captured signal using the 4-by-4 matrix of EPIC sensors.

In this trial we were able to obtain the signal with two limbs of the mouse in contact with two sensors. Figure 15 shows the result after notch filtering the raw signal.

**Figure 16:**
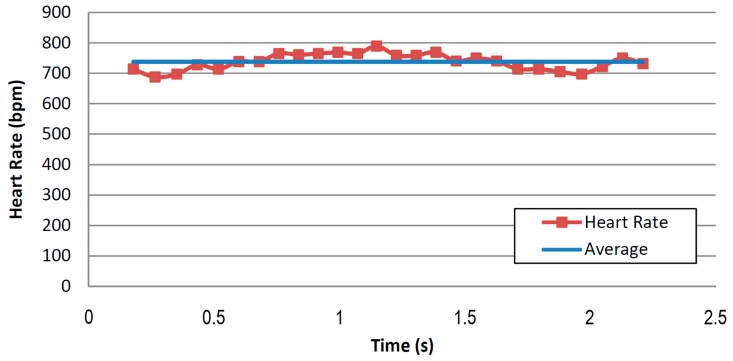
Calculation of the instantaneous heart rate based on the signal peaks series.

The signal quality, despite being far from excellent, allows us to find some patterns similar to those of the QRS complex (Combination of the Q, R and S waves) and T-wave of the ECG. The instantaneous heart rate can be calculated from the time between any two consecutive events. The drawback of this method is that the calculated value can differ from the measured pulse due to variations in the heart rate associated with respiration (known as the sinus arrhythmia). Applying this method, we obtain Figure 16 which shows a very regular pattern, with an average value of 738 bpm. This value is slightly higher than those obtained using the photo-plethysmography device, due to the different area (sensor matrix) where the mouse was placed.

### 3.5. Test Trial 5: Matrix of EPIC Sensors (System C) with Connection to GND, One Paw in Contact with the Sensors

**Figure 17:**
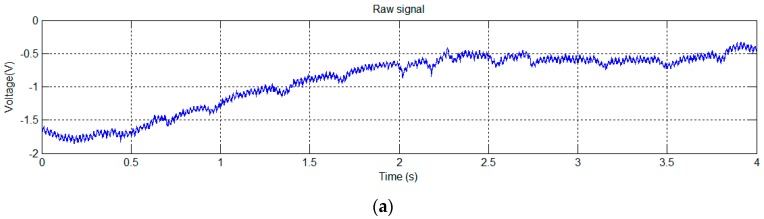

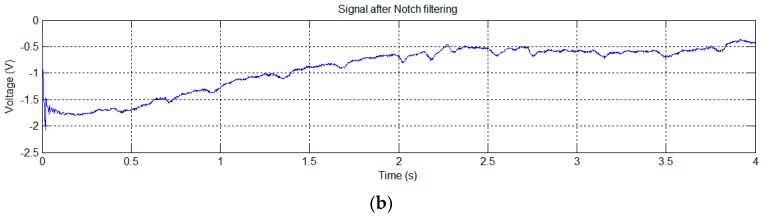
Test trial 5: signal acquired using the sensor matrix. Before (**a**) and after (**b**) the notch filter.

Figure 17 shows the captured signal before and after notch filtering. There is a slight variation in the signal, whose pattern suggests a respiratory-related signal (sinus arrhythmia). 

**Figure 18:**
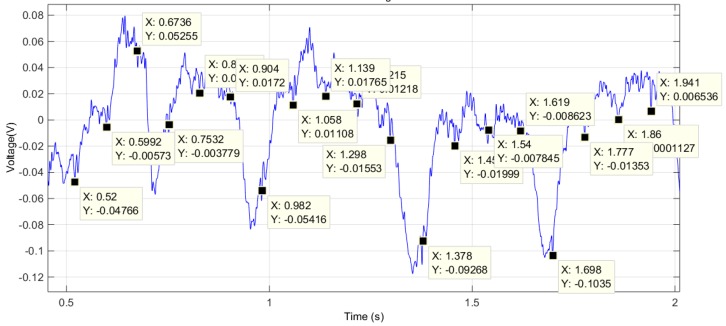
Test trial 5: signal after filtering out the variation of the midline. Lower intensity signal data collection.

By eliminating the variation of the midline and filtering the signal using a low-pass filter (first-order, cut-off frequency of 80 Hz) we get the signal shown in Figure 18. The variation of this signal is much more noticeable. We proceeded to the collection of data of the lower intensity component. This operation is done applying the same procedure as in the previous section, in order to obtain a heart rate that is fairly constant, and around 750 bpm.

## 4. Conclusions

We have developed a system using EPIC sensors to capture physiological signals that may be used to infer the level of stress to which animals are subjected.

The first contactless tests (system A) showed a strong signal that was assumed to originate in breathing. To check this, and in the absence of suitable measuring equipment, we captured video showing both the animal and the signal changes. This verified that the signal reflected the movements of the mouse. The signal corresponded to the movement of the mouse’s chest during respiration. Sometimes, sudden changes of the signal rhythm occurred, and were attributed to periods in which the animal sniffed. System A is fine for users who are satisfied with getting the breathing rate.

Once we proved the feasibility of this system for breathing rate monitoring, and due to the absence of heart-related components in our signal, we proceeded to focus on the heart rate monitoring. This proved much more challenging, due to the much smaller range of the heart rate signals. For this reason, we proceeded to change the design of the experiments, focusing on contact techniques in order to at least prove that these sensors, or similar sensors, can be used to acquire a heart rate-related signal. 

Given the impossibility of acquiring a cardiac signal using contactless techniques, we located the sensors in a platform, so that an animal placed its paws on them (system B). Because of the interesting results obtained when the animal had a leg in contact with the sensor, we used a 4-by-4 matrix of EPIC sensors (system C). This made it possible to capture a signal whose frequency was similar to that expected for mouse cardiac signals. When two of the mouse’s paws were located on two respective sensors, the captured signal showed similar shapes to those of the ECG. It is to be noted that these signals were acquired using the lower limbs of the mouse, so the shape of the ECG may be different from that expected, especially from the results familiar in humans. 

In the absence of a reference system that would allow us to capture the cardiac signals and those captured by our system simultaneously, we could not explicitly verify that both signals were related. Performing a photo-plethysmography record by using a commercial device (Vistech Systems BP-2000, Apex, NC, USA) we obtained, indirectly, data about the heart rate, which allowed us to validate the availability of heart rate–related information in the signals registered with our sensors.

If the mouse placed only one paw in contact with one of the sensors and the chest close to a second one, it was possible to obtain signals that showed both respiratory and heart rates, which could be a novel way to monitor both parameters.

If the effectiveness of this system to catch ECG in rodents is proven, there would be great advantages over current systems. There are commercial solutions that use larger conventional electrodes to capture the ECG in rodents, and these also use platforms with sensors at the bottom. However, these commercial devices require the use of conductive gels (usually, with Ag/AgCl electrodes) with the disadvantages they present.

The main disadvantage of our system is the necessity for the mouse to be positioned exactly on one or more sensors. Of course, one could try to reduce the distance between sensors, but this would cause a reduction of the measurement area. To maintain this parameter constant, the number of sensors should be increased, raising the final cost and power consumption, an important parameter for a battery-powered system.

In any case, the use of contact techniques does not allow for permanent monitoring (during transport, for example), due to the incompatibility of these techniques with the necessary housing conditions inside the containers (sawdust, urine, cleaning). For these reasons, contactless techniques are considered more useful for this purpose.

## Figures and Tables

**Table 1:** Summary of the systems tested for capture of physiological signals in rodents.

System	Name	Short Description
A	Plastic tube	There are holes for maintaining an adequate air flow. Sensors can be placed inside or outside and so can be grounded.
B	Platform	High cardboard platforms where the animal stays. Contact or contactless techniques can be used. Contact or contactless grounding can be achieved with aluminum foil.
C	Matrix of sensors	Array of 16 EPIC sensors arranged in a 4-by-4 matrix with minimal distance between them. Copper tape was used for grounding. The matrix was connected to a self-designed printed circuit board.
